# Effect of Multi-Year Environmental and Meteorological Factors on the Quality Traits of Winter Durum Wheat

**DOI:** 10.3390/plants11010113

**Published:** 2021-12-30

**Authors:** Gyula Vida, Mónika Cséplő, Marianna Rakszegi, Judit Bányai

**Affiliations:** Centre for Agricultural Research, Agricultural Institute, ELKH, 2462 Martonvasar, Hungary; cseplo.monika@atk.hu (M.C.); rakszegi.mariann@atk.hu (M.R.); banyai.judit@atk.hu (J.B.)

**Keywords:** winter durum wheat, quality, environmental effects, meteorological factors

## Abstract

A detailed study was made of the effect of rainfall, average temperature and hot days on the gluten index and Minolta b* value of winter durum wheat sown in the field in 16 consecutive crop years (2005–2020). The joint analysis of these two technological quality traits represented a complex (plant–environment–meteorological factors) approach for the identification of durum wheat cultivars carrying an optimum combination of the two traits and for the determination of quality stability. The results of GGE-biplot analysis indicated that the cultivar that had the most favorable combination of the traits was ‘MVP’, while cultivar ‘GKS’ had the best gluten strength and ‘MVH’ the best yellow pigment content. Correlation analysis and stepwise regression between various meteorological factors (rainfall, mean temperature, number of heat days per 10-day period during grain-filling) and the two technological quality traits indicated that the expected value of the quality traits could be reliably estimated based on meteorological factors, with a generally negative effect on gluten index and a positive one on yellowness in all cultivars.

## 1. Introduction

The vast majority of the durum wheat produced and marketed in the world is grown under non-irrigated, semi-arid conditions [1]. 

The main growing areas are the North and South Mediterranean zones and the Lusitanian zone [2]. Three methods are used in Europe for the production of durum wheat [3]. In countries in Southern Europe spring cultivars are sown in autumn. Due to the mild winters, the crops are unlikely to suffer cold damage, while they are able to utilize the greater quantity of precipitation falling in winter. In cooler, more northerly areas (Austria, Germany) the cultivars sown are again mostly spring types, but as the risk of freezing is greater in the colder winters, farmers sow the seed in spring. In Central and Eastern Europe (Pannonian region) most of the cultivars grown are true winter durum wheat that is sown in autumn. The main reason for this is that in this region, winter types are able to utilize the better quantities of rainfall available in autumn and winter [4], so their yield potential is substantially better than that of spring-sown crops [5,6]. 

Winter durum wheat cultivars have also been registered in the United States [7] and Canada [8], but in these countries, the ratio of winter cultivars grown is negligible compared with the spring genotypes.

Durum wheat stands are in part exposed to diverse abiotic stress factors in different environmental zones. While drought and heat stress influence plant development in the North and South Mediterranean regions, in the Pannonian zone these are combined with cold stress. The strong gluten matrix and the high content of yellow pigment are two of the most important components of the technological quality of durum wheat and both present an important breeding target for this crop [9]. While the former plays a role in determining the cooking properties of its main processing product, pasta, the latter is important in marketing. The improvement of technological quality is often linked to the development of tolerance to biotic and abiotic stress tolerance. It is known, in fact, that drought and high temperatures reduce photosynthesis and the source-to-sink transport of photosynthates in the caryopsis, thus affecting both primary and secondary metabolism [10]. The damaging effect of high temperature on gluten quality was reported almost 100 years ago in bread wheat. Observations made at that time showed that the protein content rise when the temperature was higher than average at the ripening stage, but this was not associated with an improvement in gluten quality [11]. The heat shock caused a significant negative effect on individual grain dry mass, but a positive effect on flour protein concentration and relative glutenin macropolymer content and average particle size [12]. According to Finney and Fryer [13], heat stress in the last 15 days of the ripening process led to a decrease in the loaf volume. Blumenthal et al. [14] attributed the unfavorable change in gluten quality to an increase in the gliadin:glutenin ratio. Dupont and Altenbach [15] published a review of how environmental factors influenced morphological traits and physiological processes during the grain-filling period of bread wheat, including technological quality via the synthesis of storage proteins.

When Ames et al. [16] studied ten durum wheat cultivars in eight environments for traits related to gluten strength, a significant genotype × environment effect for the gluten index and the SDS sedimentation value was detected, with a prevalence of the genotypic one, on both parameters. Heat stress in the late phase of the grain-filling period does not affect the yield or the protein quantity, but may still spoil the pasta-making quality due to ‘dough weakening’ [17]. As the ripening process proceeds, there is a gradual reduction in the gliadin:glutenin ratio, with a parallel increase in the gluten index. As the temperature rises, the gluten index also increases up to approximately 30 °C, but this trend is reversed at higher temperatures, where a weakening of the gluten can be observed [18]. 

In studies carried out in Spain, Rharrabti et al. [19] found that a higher mean temperature led to a rise in the yellow pigment content, while drought improved the gluten strength estimated by SDS sedimentation volume. However, in experiments in Portugal, the environment had no significant effect on these quality traits [20], which could have been due to the different environmental zone [2]. In experiments performed by CIMMYT (Mexico) the quality recorded on the basis of lactose retention capacity (LARC) and mixograph midline peak time (MPT), which are related to gluten strength, was better after drought stress and poorer after heat stress, while the Minolta b* value and the swelling index of glutenin (SIG) rose in response to both types of stress [21]. In another experiment, intense heat stress reduced not only the physical parameters of the cereal grains (test weight and thousand-kernel weight) but also the alveograph W and Minolta b* values, while the protein and zinc contents were observed to rise [22]. A recent experiment at CIMMYT also made it clear that drought had a favorable influence on the technological quality of durum wheat [22], as confirmed by experiments conducted in Turkey: the SDS sedimentation value, which is correlated to gluten strength, was 20.2% higher in the grain of plots exposed to drought than in the irrigated control plots [23].

The countries, where winter cultivars are grown, have less extreme rainfall and temperature conditions during the grain-filling period than Mediterranean countries. Nevertheless, the year has been found to have an effect on technological quality. In fact, under Hungarian conditions, the gluten spread, a trait related to gluten strength, and the yellow pigment content of the semolina exhibited substantial changes in different years [24,25]. In Serbia, the hot dry weather during the grain-filling period in 2009 and the cool wet weather in 2010 led to significant reductions in the protein and wet gluten contents, though no change was observed in the yellow pigment content [26]. In Ottava, too, where a typically Mediterranean climate prevails, the gluten index decreased in an environment with temperatures between 30 and 40 °C [18]. Water withdrawal led to a significant increase in the protein content of cereal cultivars [27], while in response to heat stress treatment there was a decline in the gliadin:glutenin ratio of the samples and in the ratio of the unextractable polymeric protein fractions [28]. When heat stress treatment was applied from the 12th day after heading for a period of 15 days, the bread wheat cultivar investigated had a significantly lower Zeleny sedimentation value, but in the same experiment, there was a significant increase in this trait for the winter durum wheat cultivar ‘Mv Makaróni’ [29]. 

Based on this, in the present work, five winter durum wheat cultivars were sown under field conditions between 2005 and 2020 in order to carry out a complex analysis of plant–environment–meteorological factors aimed at obtaining an accurate picture of the magnitude of changes in individual parameters and of the effect of environmental and meteorological factors on the quality traits of winter durum wheat. The analysis of performance using data from multi-year trials allowed a comprehensive, comparative understanding of the germplasm evaluated and of the crop years when the trials were conducted [30]. The specific aims were (1) to explore the effect of the year on the gluten index and Minolta b* value of winter durum wheat cultivars, (2) to evaluate the genotypes for both quality traits and their stability across environments, (3) to identify meteorological factors with a decisive effect on gluten index and Minolta b* value, and (4) to analyze the combined effect of meteorological factors on the gluten index and Minolta b* values of the cultivars tested.

## 2. Results

### 2.1. Effect of the Genotype, the Crop Years and Their Interaction on the Gluten Index and Minolta b* Values of Winter Durum Wheat Cultivars, Based on 16 Consecutive Crop Years 

In the statistical analysis, significant differences were demonstrated between years and durum wheat genotypes on the basis of the critical values for F distribution at the relevant probability levels and degrees of freedom. ANOVA analysis showed the significant effect of the crop years, the genotype and their interaction on gluten index and Minolta b* values, based on the critical values for F distribution at the relevant probability levels and degrees of freedom (Table 1).

The influence of the crop years on the gluten index and Minolta b* values of five winter durum wheat cultivars (‘GKB’, ‘GKS’, ‘MVM’, ‘MVP’ and ‘MVH’) grown in 16 consecutive years (2005–2020) was shown in Table 2, leading to the identification of the meteorological factors having a decisive effect on these traits.

Judging from Tukey’s HSD test, the 2008/2009 growing season was the most favorable for the development of a strong gluten matrix (mean gluten index value = 89.81) (Table 2). The lowest gluten index was recorded in 2019 (27.48), though the value obtained in 2012 did not differ significantly from this (34.00). Large significant differences were found between the genotypes: “GKS” and “MVP” had the highest and most stable gluten index values, while the “MVM” variety had the lowest in each crop year. The Minolta b* values ranged from 21.42 to 28.14 in the different crop years, the least favorable crop year was clearly seen to be 2017 (21.42). All the cultivars with the exception of ‘GKS’ (24.18) and ‘MVM’ (24.39) had significantly different Minolta b* values. ‘MVH’ was ranked first, with an outstandingly high value (27.19), followed by ‘MVP’ (24.95), while the cultivar with the smallest mean value was ‘GKB’ (22.51). 

### 2.2. Identifying Superior Durum Wheat Genotypes Based on the Gluten Index and Minolta b* Values 

**Figure 1:**
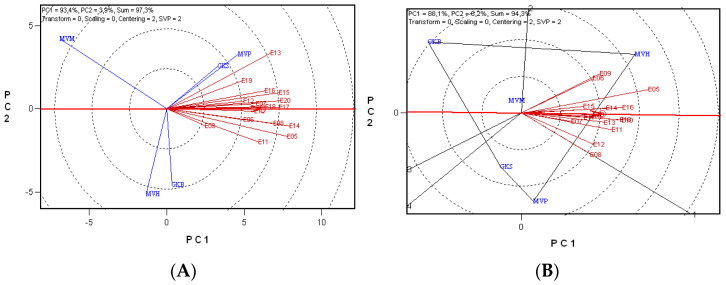
Polygon view of the GGE biplots (‘which-won-where’ model) for five durum wheat cultivars based on the gluten index (**A**), or on the Minolta b* value (**B**) over 16 years. Environment-centred (Centering = 2) GGE biplot analysis based on non-scaled data (Scaling = 0) with environment-metric (SVP = 2) plotting. E05–E20: years 2005–2020; ‘GKB’, ‘GKS’, ‘MVM’, ‘MVP’, ‘MVH’: genotypes; PC1, PC2: principal component variables; environment vectors are plotted with thin and the average environment axis (AEA) with a thick red line; O: year mean.

The applied method of principal component analysis made it possible to investigate the correlations between the examined crop years. The method is used for the analysis of object diversity in regard to quality traits of the winter durum wheat. The most recent method, the GGE biplot model, provided a complete visual evaluation of all aspects of the data by creating a biplot that simultaneously represents both mean performance and stability [31]. The first two principal components, PC1 and PC2, accounted for 93.40% and 3.90% of the GGE sum of squares, respectively, explaining a total of 97.30% of the variation (Figure 1A) and revealing a differential gluten index performance between the durum wheat genotypes across the years due to the presence of a genotype × environment interaction (G × E). The length of the vectors representing the various years differed, but in all cases, the angle they enclosed was much less than 90°, showing that the correlation between the years was close and positive. The angles between the vectors and the average environment axis were indicative of whether a year could be regarded as having a ‘representative’ or ‘special’ effect. On this basis, 2013 was the clearest example of a ‘special’ year, while 2007, 2010 and 2018 were located closest to the average. The length of the vectors indicates the extent to which each year made it possible to differentiate between the genotypes. The E14 and E05 vectors were the longest, showing that the durum wheat cultivars could be differentiated to the greatest extent on the basis of their gluten indices in 2005 and 2014. By contrast, the gluten index values of the cultivars differed to the least extent in 2009 (E09). It is clear from the results presented in Table 2, that the highest gluten index values were recorded this year and that the percentage differences between the cultivars were smaller than in the other years. The ‘which-won-where’ analysis makes it possible to identify the genotype with the most favorable properties for a given environment. The individual sectors represent mega-environments. As all the years were located in the same sector in Figure 1A, and as ‘MVP’ and ‘GKS’ were also found in this sector, it can be concluded that all the years had an almost identical effect on the gluten index values of the winter durum wheat cultivars tested; the cultivars with the strongest gluten matrix were ‘MVP’ and ‘GKS’, and the cultivar with the weakest gluten matrix was ‘MVM’, as this was found in the 3rd sector, on the left, at the greatest distance from the environment (year). The analysis of the Minolta b* values again revealed that a single principal component variable was dominant; PC1 and PC2 accounted for 88.10% and 6.20%, respectively, of the GGE sum of squares, explaining a total of 94.30% of the total variance (Figure 1B). It can be concluded from the acute angle enclosed by the environmental vectors that the data recorded in different crop years were positively correlated, except four years (2006, 2009, 2008, 2012), they showed the different effects on the quality trait; 2017, 2019 and 2020 were located closest to the average, the Minolta b* values of the cultivars differed to the greatest extent in 2005 and to the least extent in 2007. ‘MVH’ was the clear winner according to the analysis, being the only cultivar located in the sector containing the environmental vectors. 

**Figure 2:**
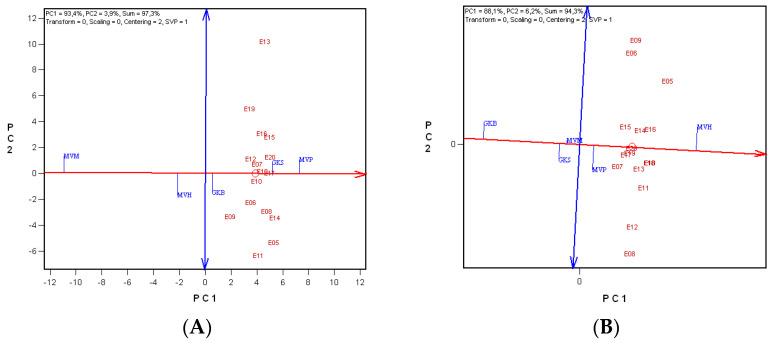
Ranking biplots based on the mean gluten index (**A**) or mean Minolta b* value (**B**) and stability of durum wheat cultivars over 16 years. Environment-centred (Centering = 2) GGE biplot analysis based on non-scaled data (Scaling = 0) with environment-metric (SVP = 2) plotting. E05–E20: years 2005–2020; ‘GKB’, ‘GKS’, ‘MVM’, ‘MVP’, ‘MVH’: genotypes; PC1, PC2: principal component variables; the average environment axis (AEA) is represented by a thick red line; values on the blue axis, supplied with arrows, represent the stability of the cultivars. The intersection of the two axes: cultivar mean; O: year mean.

The ranking biplot method was used to evaluate the genotypes on the basis of gluten index and Minolta b* value means and their stability across environments (Figure 2). The abscissa indicates higher mean values across environments and the ordinate greater variability (poorer stability) in either direction. With regard to gluten index, in all 16 years ‘MVP’ had the highest mean gluten index, followed by ‘GKS’, while ‘MVM’ had the lowest mean value (Figure 2A). Regarding stability, all of the genotypes were almost identical over an average of 16 years.

Based on the Minolta b* values of the durum wheat cultivars ‘MVH’ gave the best results, followed by ‘MVP’, ‘MVM’, ‘GKS’ and ‘GKB’. ‘MVM’, which was located almost on the average environment axis (AEA), was extremely stable, averaged over 16 years (Figure 2B).

#### Interaction between the Years and Winter Durum Wheat Cultivars on the Basis of Gluten Index and Minolta b* Data over 16 Years

In the course of selection, breeders need to select simultaneously for several traits, the aim being to develop cultivars with the best combination of these traits. GGE biplot analysis makes it possible to investigate several traits at the same time.

**Figure 3:**
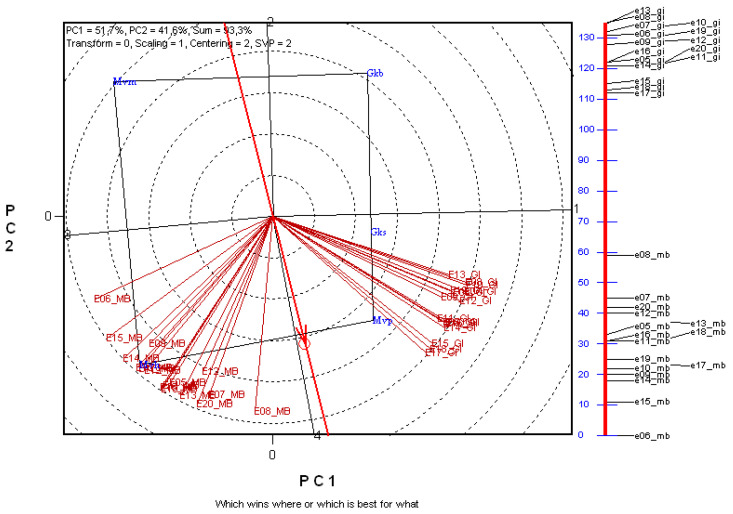
Correlations between years and winter durum wheat cultivars on the basis of gluten index (GI) and Minolta b* (MB) data over 16 years. Environment-centred (Centering = 2) GGE biplot analysis based on non-scaled data (Scaling = 0) with environment-metric (SVP = 2) plotting. E05–E20: years 2005–2020; ‘Gkb’, ‘Gks’, ‘Mvm’, ‘Mvp’, ‘Mvh’: genotypes; PC1, PC2: principal component variables; environment vectors are plotted with thin and the average environment axis (AEA) with a thick red line; O: year mean. The linear map of the environment vectors is shown on the right of the figure, where the scale represents the angle enclosed by the vectors, expressed as °.

The calculations revealed that two principal component variables had eigenvalues exceeding 1, and these explained 93.3% of the complete variance of the system (Figure 3). The gluten index and Minolta b* vectors formed two separate groups, with an approximately 90° angle between the mean vectors of the groups, indicating the independence of the two traits. The cultivars located at the corners of the rectangle had different combinations of the two technological quality traits tested. ‘MVP’ was located in the same sector as the gluten index vectors, at one corner of the rectangle, indicating that the gluten index of this cultivar was outstanding. ‘MVH’ was in a similar relationship with the Minolta b* vectors. ‘MVM’ was in the sector opposite the gluten index vectors, indicating a weak gluten matrix, while ‘GKB’ occupied a similarly unfavorable position with respect to the Minolta b* vectors. 

**Figure 4:**
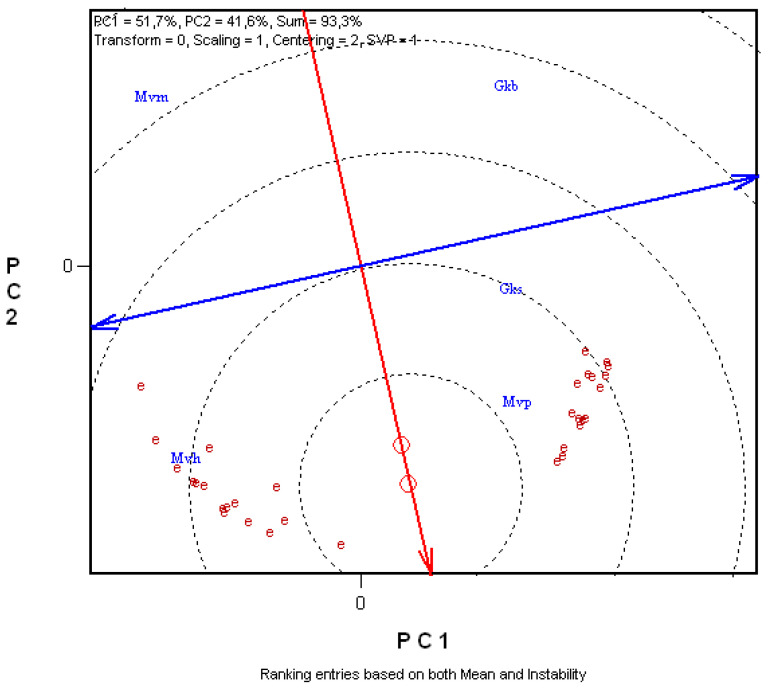
Comparison between the genotypes tested and the ideal genotype on the basis of gluten index (right sector) and Minolta b* values (left sector) over 16 years. Environment-centred (Centering = 2) GGE biplot analysis based on non-scaled data (Scaling = 0) with environment-metric (SVP = 2) plotting. E05–E20: years 2005–2020; ‘Gkb’, ‘Gks’, ‘Mvm’, ‘Mvp’, ‘Mvh’: genotypes; PC1, PC2: principal component variables; the average environment axis (AEA) is represented by a thick red line; the stability axis is plotted with thick blue line; O: year mean; O in the middle of the concentric circles: ideal genotype.

Figure 4 allows the durum wheat cultivar with the best approximation to the ‘ideal’ genotype (in this case, with the best combination of the given traits) to be identified. The value of a genotype is determined by its distance from the theoretical genotype located in the middle of the concentric circles (best for all traits, with perfect stability). The closer the genotype is to this point, the better the combination of desired traits. According to this analysis, ‘MVP’ came closest to the ideal genotype, with the best combination of these traits. ‘GKS’ and ‘MVH’ were at approximately the same distance from the ideal, the former excelling chiefly for gluten strength, the latter for yellow pigment content. ‘MVM’ and ‘GKB’ was located at the greatest distance from the ideal genotype. 

### 2.3. Identification of the Meteorological Factors Determining the Gluten Index and Minolta b* Values

The correlation analysis was performed on a data matrix that provided for 61 meteorological factors (26 rainfall + 23 mean temperature + 12 heat day data) and five cultivars (cultivar mean × 2 traits) (Table S1). The significant factors for both parameters were shown in Table 3. 

The gluten index data measured for each cultivar and their mean were negatively influenced by the majority of meteorological data with identified effects (Table 3). The increased mean monthly temperature in June and the mean temperature in the first 10 days of July significantly decreased the gluten index for all the durum wheat cultivars and for the cultivar mean. An increase in the number of heat days in the middle 10 days of May had a positive significant effect on the gluten index of ‘MVM’ (r = 0.859 ***), ‘GKB’ (r = 0.533 *), ‘MVH’ (r = 0.511 *) and on the cultivar mean (r = 0.617 *). The gluten strength of two cultivars (‘MVP’ and ‘MVH’) and the cultivar mean significantly decreased in response to greater rainfall in the first 10 days of April, however, ‘MVM’, which had a weak gluten matrix, responded favorably to the rainfall quantity in the third week of June (r = 0.566 *). Cultivar-specific effects were detected for the mean temperature in the first 10 days of June in ‘GKS’, and for the quantity of rainfall in November, and in the last 10 days of May and in the whole of May, and the number of heat days in the middle 10 days of June in ‘MVP’.

The Minolta b* (MB) value was significantly influenced by seven meteorological factors. The number of heat days in the last 10 days of May intensified the yellow color of all the cultivars and the cultivar mean (r ranged from 0.568 * to 0.777 ***), and a similar effect was observed for the total number of heat days in May, in June and throughout the vegetation period. Rainfall at the beginning of June also had a favorable effect on the intensity of the yellow color (r ranged from 0.546 * to 0.623 **). A cultivar-specific effect was only detected for ‘MVH’, in which the Minolta b* value was enhanced by the quantity of rainfall in the last 10 days of April, but reduced by the mean temperature in October.

### 2.4. Combined Effect of Meteorological Factors on the Gluten Index and Minolta b* Values of the Cultivars Tested 

In the calculations, the rainfall (total rainfall in the months from August to March, quantity of rainfall every 10 days from April to harvest), the mean temperature data (monthly data from October to March, 10-day data from April onwards) and the number of heat days (every 10 days from early May to harvest) were taken as independent variables. The gluten index (gluten strength) was diversely influenced by meteorological factors in the different cultivars. The most accurate regression function could be compiled using only two factors for ‘GKS’, while four were needed for ‘MVM’, seven for ‘MVP’ and ‘MVH’ and nine for ‘GKB’. When calculations were made for the average of the five cultivars, four factors played a decisive role in the development of the gluten index (Table 4).

As several of the winter durum wheat cultivars gave an excellent approximation to the expected value, but the functions were extremely complicated (World S1), data are also given for models where the value of the multiple coefficients of determination (R^2^) exceeded 0.9. In the latter case the expected value of the gluten index can be estimated to a satisfactory approximation using a simplified function:

(aa) ‘GKB’ gluten index = 336.608 − 6.739 × mean temperature in 2nd 10 days of June − 12.798 × No. of heat days in 2nd 10 days of May − 5.758 × mean temperature in 1st 10 days of July − 0.310 × October rainfall − 1.956 × mean temperature in February
(bb) ‘GKS’ gluten index: cannot be simplified
(cc) ‘MVM’ gluten index = 44.806 + 28.670 × No. of heat days in 2nd 10 days of May − 4.098 × mean temperature in October + 0.195 × December rainfall
(dd) ‘MVP’ gluten index = 120.549 − 0.792 × rainfall in 1st 10 days of April − 2.047 × No. of heat days in 2nd 10 days of June + 0.358 × rainfall in 1st 10 days of July + 6.024 × No. of heat days in 2nd 10 days of May − 1.414 × mean temperature in 1st 10 days of July
(ee) ‘MVH’ gluten index = 239.199 − 1.445 × rainfall in 1st 10 days of April − 3.787 × mean temperature in February − 5.224 × mean temperature in 2nd 10 days of June + 12.551 × No. of heat days in 2nd 10 days of May − 3.313 × mean temperature in 1st 10 days of July
(ff) Mean gluten index for the five cultivars: cannot be simplified.

The Minolta b* value was diversely influenced by meteorological factors in the different cultivars (World S1), except ‘MVM’ and ‘MVP’ genotypes. The regression function could be compiled using only one factor for them, as ‘No. of heat days in 3rd 10 days of May’ played a decisive role in the development of Minolta b* value. Moreover, this factor was determinant in all varieties. The most accurate regression function could be compiled using four factors for ‘GKB’, five factors for ‘GKS’ and fifteen for ‘MVH’. When calculations were made for the average of the five cultivars, eleven factors played a decisive role in the development of the Minolta b* value (Table 5).

Simplified functions are suitable for the estimation of Minolta b* values

(εε) ‘MVH’ Minolta b* = 31.523 + 0.079 × rainfall in 1st 10 days of June − 0.489 × mean temperature in 1st 10 days of June + 0.615 × mean temperature in October − 0.228 × mean temperature in 1st 10 days of May
(φφ) Average Minolta b* for the five cultivars = 27.603 + 0.575 × No. of heat days in 3rd 10 days of May − 0.459 × mean temperature in 1st 10 days of June + 0.333 × mean temperature in 3rd 10 days of April + 0.032 × August rainfall − 0.020 × September rainfall

Stepwise regression analysis made it possible to identify meteorological factors with a decisive influence on the genotype-dependent and mean gluten index and Minolta b* value. Averaged over the cultivars, the number of heat days in the second 10 days of May and the mean temperature in the final phase of grain filling were of outstanding importance for the gluten index. Heat days had a positive influence on gluten strength in the early stages of development, and a negative effect in later stages. The negative effect of the precipitation quantity in October can be explained by delays in sowing. Hot weather soon after pollination (No. of heat days in the last 10 days of May) had a positive influence on the Minolta b* value, but in later stages (mean temperature in the first 10 days of June) the effect became negative.

When the paired t-test was used to analyze the similarity of the estimated data (substituted into the gluten index and Minolta b* functions) and the original data set (Table 6), the two sets of data were found to agree at a high level of probability (*p* > 0.94) for all the durum wheat cultivars and for the cultivar mean. 

As the five genotypes differ as regards both gluten index and Minolta b* value, it can be concluded from the R^2^ values of the regression functions calculated from the cultivar mean for gluten index (0.873) and Minolta b* value (1.000) that the expected values of these important technological quality traits can be reliably estimated on the basis of meteorological factors. The functions for the calculation of the gluten index include data not available until the end of the first 10 days in July, so early estimation is not possible for this trait. The Minolta b* value, on the other hand, is determined by the first ten days in June, so it is possible to predict with considerable certainty 3–4 weeks before harvest. 

## 3. Discussion

The important objective of the winter durum wheat improvement program is to develop not only heat, drought and frost tolerant, high yielding with better quality but also stable varieties. It was hypothesized that it should be possible to identify meteorological factors with an above-average influence on the gluten index and Minolta b* values of winter durum wheat cultivars. In order to prove this, the correlation between various meteorological factors (monthly total precipitation and mean temperature from August to March, the same factors for each 10-day period from April to harvest, and the number of heat days in each 10-day period in the grain-filling period) and two technological quality parameters was analyzed by means of correlation analysis and stepwise regression analysis.

In the present experiment, the cultivar with the strongest gluten matrix was more stable than those with weak gluten, exhibiting less change in the gluten index in years with warmer weather during grain filling. This, therefore, confirmed in the case of winter durum wheat cultivars the observations made by Ames et al. [16] in spring genotypes. Meteorological factors related to high mean temperature or to the number of heat days are frequent terms in the equations used to predict the mean gluten index of the five cultivars. All of these are preceded by a regression coefficient with a minus sign, i.e., the higher the mean temperature at the end of the grain-filling period and/or the number of heat days during this period, the lower the gluten index and the poorer the gluten strength. 

The occurrence of several heat days in the early stages of grain filling (2nd 10 days in May) had a favorable influence on the gluten index, especially in ‘MVM’, which had a weak gluten matrix, but also in ‘GKB’ and ‘MVH’ and the cultivar mean. It can be hypothesized that if heat stress occurs during flowering (‘MVM’ is a late cultivar, generally heading in the 2nd 10 days of May), it may cause a change in the accumulation dynamics of the various types of storage proteins. Targeted experiments in the phytotron will be required to confirm this. Further studies will also be needed to decide whether the negative correlation between April precipitation quantity and the gluten index of cultivars ‘MVP’ (−0.733 **) and ‘MVH’ (−0.600 *) and the mean gluten index of the five cultivars (−0.589 *) was a direct effect or the consequence of other indirect physiological changes.

In all the cultivars, the Minolta b* value was enhanced by the number of heat days in the early stage of grain filling. Furthermore, the values of four of the five durum wheat cultivars and the cultivar mean exhibited a significant correlation with the number of heat days during the vegetation period. These results confirm the findings of Rharrabti et al. [19], who reported that higher temperatures favored an increase in yellow pigment content. The highest Minolta b* value was measured in the present work in samples taken in the driest year (2007: total rainfall quantity in the whole vegetation period: 153.8 mm), suggesting that drought improves the effect of heat stress. Similar results were found in spring durum wheat by Li et al. [21], but it was suggested that intense heat stress might reverse this process, leading to a lower Minolta b* value [22]. This conclusion was confirmed by the observations of Cseuz et al. [25] and Hadži-Tašković Šukalović et al. [26], who did not detect any significant difference between the yellow pigment content in different years under the climatic conditions of the Pannonian Region. In some cases, the year has also been known to influence this trait [24]. In experiments set up between 1991 and 1994 in Szeged (S. Hungary), a substantial difference was recorded between the yellow pigment contents detected in 1993 and 1994, with the highest value in 1994 when the weather was cooler (17.97 °C), with more rainfall (62.6 mm) during the grain-filling period (May and June), with respect to 1993, warmer (mean temperature: 19.38 °C) and slightly drier (56.9 mm). This suggests that cooler weather with more rainfall in the early stage of grain filling had a favorable effect on the yellow pigment content. In Martonvásár (Hungary) durum wheat reaches this phenophase a few days later than in south Hungary, as it is located 200 km north of Szeged. In conclusion, the present data showed the favorable effect of a larger quantity of rainfall in the first 10 days of June, when the early stage of grain filling occurred. The present data thus confirm those of Matuz et al. [24] as to the favorable effect of rainfall during early grain filling. 

## 4. Conclusions

Analyzing correlations by means of stepwise regression analysis proved to be a satisfactory method for the joint detection of the effect of various factors. GGE biplot analysis on the effect of the year on five winter durum wheat cultivars in 16 growing seasons revealed the years (environments) when selection could best be performed for gluten index and Minolta b*. The cultivars tested differed considerably for both yellow pigment content and gluten strength. The gluten index was outstandingly high for the cultivar ‘MVP’, followed by ‘GKS’, which also had excellent stability, whereas ‘MVH’ was ranked first for Minolta b*, but had poor stability. Although yellow pigment production was determined jointly by a number of meteorological factors, the final value of Minolta b* could be predicted well before full ripening for the majority of durum wheat cultivars. ‘MVP’ was the best approximation to the ideal genotype (a theoretical cultivar with the optimum combination of the two traits), so on the basis of the long-term database, this cultivar can be recommended for use as a crossing partner in breeding.

As reported by other authors, analyzing correlations by means of stepwise regression analysis proved to be a satisfactory method for the joint detection of the effect of various factors. 

## 5. Materials and Methods

### 5.1. Plant Materials

Five genotypes of winter durum wheat (*T. turgidum* ssp. *durum*) were evaluated under rain-fed conditions in the nursery of the Centre for Agricultural Research in Martonvásár, Hungary in the autumn cropping seasons of 2005–2020.

The cultivars used as standards in state cultivar trials (‘GKB’ and ‘GKS’) were tested, together with three Martonvásár cultivars (‘MVM’, ‘MVP’ and ‘MVH’) bred and cultivated in the Carpathian Basin. 

### 5.2. Site Description and Set-Up for the Field Experiment

The rain-fed experiment was arranged in a randomized complete block design with three replications and a sowing density of 500 seeds/m^2^. The geo-coordinates of the field were latitude 47°30′41.70″ N and longitude 18°81′83.34″ E (Martonvásár, Hungary). The genotypes were sown in the middle of October each year. The soil texture was loam with good P and K supplies and good water permeability. Individual plots measured 0.96 m × 6 m and consisted of eight rows, 12 cm apart. Nutrients were supplied in autumn at a rate of 60:60:60 kg ha^−1^ N:P:K active ingredients, followed by topdressing with 60 kg ha^−1^ N in early spring. No fungicide treatment was applied during the vegetation period, and weeds and pests were controlled chemically. The plots were harvested at full maturity using a small-plot combine harvester.

The meteorological data used for the analysis were obtained from the database of an automatic weather station located in Martonvásár, Hungary (Table 7). The data collected were the precipitation (mm) during the whole vegetation period and specifically during grain filling; mean temperatures (°C) during the vegetation period and the grain-filling period; the number of heat days (maximum daily temperature ≥30 °C); and the meteorological stress factors characteristic of the vegetation period.

### 5.3. Determination of Gluten Index and Minolta b* Values

Gluten index and b* values of the winter durum wheat samples were determined on semolina produced using a Chopin CD2 mill and purified with a Chopin Laboratory Purifier according to the ICC158 standard (ICC, 1995). The gluten index was determined using a Perten Glutomatic 2200 instrument and a Perten 2015 Centrifuge (Perten Instruments AB, Hägersten, Sweden). The Minolta b* values were determined using first a Minolta CR-300 and from 2016 onwards a Minolta CR-400 chromameter (Minolta Camera Co. Ltd., Osaka, Japan). 

### 5.4. Statistical Analysis

Analysis of variance was performed for years and cultivars using the one-factor random block design model combined with the year in the MSTAT-C software package (Michigan State University, East Lansing, MI, USA). The significance of differences between the mean values of the main factors was determined using Tukey’s HSD test. Complex correlations between the combined effects of several factors were analyzed by means of stepwise regression analysis. Interactions and correlations between years and cultivars were determined using principal component analysis, while the best-performing genotype was selected by means of principal component biplot analysis (GGEbiplot Pattern Explorer, Version 8.1) [31]. 

## Figures and Tables

**Table 1:** Analysis of variance of gluten index and Minolta b* values for five winter durum wheat cultivars in 2005–2020.

Factor	Df	SQ	MQ	F-Value	Significance
	Gluten index				
Year	15	33,843.251	2256.217	46.893	0.000
Error (a)	16	769.834	48.115		
Genotype	4	99,461.842	24,865.46	1144.469	0.000
Year × Genotype	60	12,062	201.033	9.253	0.000
Error (b)	64	1390.504	21.727		
Total	159	147,527.431			
	Minolta b*				
Year	15	605.44	40.363	560.278	0.000
Error (a)	16	1.153	0.072		
Genotype	4	364.886	91.222	767.01	0.000
Year × Genotype	60	70.622	1.177	9.897	0.000
Error (b)	64	7.612	0.119		
Total	149	1049.712			

Df—degrees of freedom, SQ—sum of squares, MQ—mean square.

**Table 2:** Mean values of the gluten index and Minolta b* values of winter durum wheat cultivars (Martonvásár, 2005–2020).

Year	‘GKB’		‘GKS’		‘MVM’		‘MVP’		‘MVH’		Cultivar Mean	
	GI	MB	GI	MB	GI	MB	GI	MB	GI	MB	GI	MB
2004/2005	72.74	24.73	87.63	26.11	1.89	25.72	91.44	27.06	62.49	31.80	^bc^63.24	^c^27.08
2005/2006	75.08	26.18	91.73	27.13	29.78	28.40	79.73	26.37	61.60	30.51	^bc^67.58	^ab^27.72
2006/2007	67.27	26.97	83.88	27.28	22.82	27.60	85.86	29.24	48.01	29.63	^bcd^61.57	^a^28.14
2007/2008	77.72	24.96	82.02	27.85	10.79	26.43	88.13	29.03	48.04	28.99	^bcd^61.34	^bc^27.45
2008/2009	98.22	22.60	93.71	22.63	68.54	22.85	98.61	22.71	89.95	26.91	^a^89.81	^ef^23.54
2009/2010	64.86	20.84	76.72	23.16	15.95	24.26	77.63	24.36	42.92	26.76	^cde^55.62	^e^23.88
2010/2011	83.34	20.01	80.53	22.05	21.43	22.72	94.36	23.80	72.15	25.23	^b^70.36	^gh^22.76
2011/2012	37.48	20.41	48.67	24.72	1.67	23.17	59.69	23.53	22.48	25.12	^gh^34.00	^f^23.39
2012/2013	31.41	22.64	78.58	25.18	7.53	24.38	74.46	25.28	9.90	27.57	^fg^40.38	^d^25.01
2013/2014	66.87	22.53	87.61	24.31	1.76	25.21	93.16	24.81	58.88	27.56	^bcd^61.66	^d^24.88
2014/2015	39.42	21.47	69.82	22.50	1.64	23.80	90.21	23.15	41.20	25.14	^ef^48.46	^fg^23.21
2015/2016	42.65	20.06	69.67	21.72	7.58	21.79	80.88	22.72	35.73	25.80	^ef^47.30	^h^22.42
2016/2017	47.29	19.60	80.52	20.71	3.97	21.64	90.05	21.93	55.30	23.24	^cde^55.43	^i^21.42
2017/2018	43.10	21.20	65.24	23.60	3.75	23.58	82.45	24.40	45.99	26.96	^ef^48.11	^e^23.95
2018/2019	26.01	22.80	39.75	23.70	1.50	24.81	60.77	25.37	9.37	26.68	^h^27.48	^d^24.67
2019/2020	50.81	23.27	69.60	24.23	1.77	23.87	90.61	25.49	39.28	27.18	^def^50.41	^d^24.81
Average	^C^57.77	^d^22.51	^B^75.36	^c^24.18	^C^12.65	^c^24.39	^A^83.63	^b^24.95	^D^46.46	^a^27.19	55.17	24.64
Minimum	26.01	19.60	39.75	20.71	1.50	21.64	59.69	21.93	9.37	23.24	27.48	21.42
Maximum	98.22	26.97	93.71	27.85	68.54	28.40	98.61	29.24	89.95	31.80	89.81	28.14
St. Dev.	20.80	2.25	14.76	2.10	17.39	1.92	11.22	2.13	21.24	2.19	15.02	2.01

GI = Gluten index. MB = Minolta b* value, St. Dev. = Standard deviation. For each quality parameter, different letters indicate significant differences among years (in columns) and cultivar (in rows), based on Tukey’s HSD test (*p* < 0.05).

**Table 3:** Pearson’s correlation coefficients between meteorological factors and the gluten index and Minolta b* values of the five durum wheat genotypes over 16 years. *, ** and *** denote significant differences at the *p* < 0.05, 0.01 and 0.001 levels of probability.

Meteorological Factor	‘GKB’	‘GKS’	‘MVM’	‘MVP’	‘MVH’	Cultivar Mean
	Gluten Index					
November rainfall				0.517 *		
Rainfall, 1st 10 days of April				−0.733 **	−0.600 **	−0.589 *
Rainfall, 3rd 10 days of May				−0.517 *		
May rainfall				−0.497 *		
Rainfall, 3rd 10 days of June			0.566 *			
Mean temp., 1st 10 days of June		−0.502 *				
Mean temp., 2nd 10 days of June	−0.535 *			−0.623 *	−0.586 *	−0.528 *
Mean temperature in June	−0.556 *	−0.660 **		−0.557 *	−0.546 *	−0.615*
Mean temp. 1st 10 days of July	−0.517 *	−0.616 *		−0.592 *	−0.498 *	−0.545 *
Heat days, 2nd 10 days of May	0.533 *		0.859 ***		0.511 *	0.617 *
Heat days, 2nd 10 days of June				−0.544 *		
	Minolta b*					
Rainfall, 3rd 10 days of April					0.561 *	
Rainfall 1st 10 days of June		0.583 *	0.546 *	0.617 *	0.623 *	0.597 *
Mean temperature in October					−0.607 *	
Heat days, 3rd 10 days of May	0.671 **	0.626 *	0.568 *	0.777 ***	0.609 *	0.688 **
Heat days in May	0.617 **	0.586 *		0.663 **	0.593 *	0.622 *
Heat days in June	0.520 *	0.503 *	0.583 *	0.536 *		
Total no. of heat days	0.662 **	0.611 *	0.728 **	0.703 **		0.670 **

**Table 4:** Characteristics of the gluten index functions developed by stepwise regression.

Cultivar	Model	No. of Meteorological Factors in the Equation	Code	Multiple Coefficient of Determination (R²)
‘GKB’	a	9	Jun2T, May2H, Jul1T, OctR, FebT, Jun2H, OctT, May2R, Jun3R	0.999
	aa	5	Jun2T, May2H, Jul1T, OctR, FebT	0.924
‘GKS’	b	2	Jul1T, May1R	0.636
‘MVM’	c	4	May2H, OctT, DecR, Jul1T	0.935
	cc	3	May2H, OctT, DecR	0.905
‘MVP’	d	7	Apr1R, Jun2H, Jul1R, May2H, Jul1T, May1R, Apr3R	0.988
	dd	5	Apr1R, Jun2H, Jul1R, May2H, Jul1T	0.942
‘MVH’	e	7	Apr1R, FebT, Jun2T, May2H, Jul1T, MarT, AugR	0.980
	ee	5	Apr1R, FebT, Jun2T, May2H, Jul1T	0.912
Cultivar Mean	f	4	May2H, Jun2T, Jul1T, OctR	0.873

Note: Jun2T: mean temperature in 2nd 10 days of June, May2H: No. of heat days in 2nd 10 days of May, Jul1T: mean temperature in 1st 10 days of July, OctR: October rainfall, FebT: mean temperature in February, Jun2H: No. of heat days in 2nd 10 days of June, OctT: mean temperature in October, May2R: rainfall in 2nd 10 days of May, Jun3R: rainfall in 3rd 10 days of June, May1R: rainfall in 1st 10 days of May, DecR: December rainfall, Apr1R: rainfall in 1st 10 days of April, Jul1R: rainfall in 1st 10 days of July, Apr3R: rainfall in 3rd 10 days of April, MarT: mean temperature in March, AugR: August rainfall. Abbreviations for meteorological factors: 3-letter month code + 1, 2, 3 (1st, 2nd, 3rd 10-day period) or nothing in the case of the whole month + R = rainfall, T = mean temperature, H = No. of heat days.

**Table 5:** Characteristics of the Minolta b* functions developed by stepwise regression.

Cultivar	Model	No. of Meteorological	Code	Multiple Coefficient
		factors in the equation		of determination (R²)
‘GKB’	α	4	May3H, May3T, Apr3R, Jun3R	0.867
‘GKS’	β	5	May3H, Apr3T, Jun3H, Jun3R, Jun1T	0.885
‘MVM’	λ	1	May3H	0.325
‘MVP’	δ	1	May3H	0.602
‘MVH’	ε	15	Jun1R, Jun1T, OctT, May1T, JanT, Jul1R, DecT, Jul1T, JanR, SepR, May3H, AugR, Jun2R, Apr2R, MarT	1.000
	εε	4	Jun1R, Jun1T, OctT, May1T	0.908
Cultivar Mean	Φ	11	May3H, Jun1T, Apr3T, AugR, SepR, May1R, DecR, FebT, May3R, Apr1T, May2T	1.000
	ΦΦ	5	May3H, Jun1T, Apr3T, AugR, SepR	0.915

Note: May3H: No. of heat days in 3rd 10 days of May, May3T: mean temperature in 3rd 10 days of May, Apr3R: rainfall in 3rd 10 days of April, Jun3R: rainfall in 3rd 10 days of June, Apr3T: mean temperature in 3rd 10 days of April, Jun3H: No. of heat days in 3rd 10 days of June, Jun1T: mean temperature in 1st 10 days of June, Jun1R: rainfall in 1st 10 days of June, OctT: mean temperature in October, May1T: mean temperature in 1st 10 days of May, JanT: mean temperature in January, Jul1R: rainfall in 1st 10 days of July, DecT: mean temperature in December, Jul1T: mean temperature in 1st 10 days of July, JanR: January rainfall, SepR: September rainfall, AugR: August rainfall, Jun2R: rainfall in 2nd 10 days of June, Apr2R: rainfall in 2nd 10 days of April, MarT: mean temperature in March, May1R: rainfall in 1st 10 days of May, DecR: December rainfall, FebT: mean temperature in February, May3R: rainfall in 3rd 10 days of May, Apr1T: mean temperature in 1st 10 days of April, May2T: mean temperature in 2nd 10 days of May. Abbreviations for meteorological factors: 3-letter month code + 1, 2, 3 (1st, 2nd, 3rd 10-day period) or nothing in the case of the whole month + R = rainfall, T = mean temperature, H = No. of heat days.

**Table 6:** Test of the similarity of the original data set and that estimated using regression functions using a paired *t*-test.

Gluten Index			Minolta b*		
Function ^1^	*t*-Value	Probability ^2^	Function ^1^	*t*-Value	Probability ^2^
a	−0.02899	0.97726	α	−0.06414	0.94970
aa	−0.01010	0.99208	β	−0.00513	0.99765
b	−0.00299	0.99765	λ	0.00190	0.99851
c	0.01959	0.98463	δ	0.00074	0.99942
cc	−0.00155	0.99878	ε	0.01055	0.99173
d	−0.03538	0.97225	εε	−0.09425	0.92616
dd	0.00102	0.99920	Φ	0.03005	0.97642
e	−0.03542	0.97221	ΦΦ	0.04009	0.96855
ee	−0.00579	0.99546			
f	−0.00687	0.99461			

^1^ See previous text for model descriptions; ^2^ probability that the difference between the original and estimated data sets is 0 based on the H0 hypothesis.

**Table 7:** Meteorological characteristics of the vegetation periods between 2004/2005 and 2019/2020 (Martonvásár, Hungary).

Vegetation Period	Rainfall		Mean Temperature (°C)		No. of Heat Days ^1^	Days from January 1st		Characteristic Meteorological Stress Factor(s)
	(mm)							
	Σ	GFP	Σ	GFP		Sowing	Harvest	
2004/2005	458.4	49.2	6.96	18.69	10	280	177	Rain at harvest
2005/2006	421.6	118.4	7.30	19.01	17	283	191	Cold January, heat stress before harvest
2006/2007	167.8	86.6	10.64	20.71	30	285	173	Drought, heat stress before harvest
2007/2008	361.4	88.8	7.97	19.91	14	285	184	Above-average rainfall
2008/2009	320.0	85.5	8.32	18.06	6	283	183	Dry April and grain-filling period
2009/2010	629.5	186.5	8.06	19.96	14	281	195	Rainfall far above average
2010/2011	238.1	54.5	7.26	19.29	3	287	192	Cold December, heat stress before harvest
2011/2012	210.2	78.2	7.39	18.73	9	284	180	Drought, heat stress before harvest
2012/2013	381.8	68.2	7.63	17.96	7	279	183	Sharp cold spell in mid-March
2013/2014	304.9	87.7	9.22	17.78	6	276	183	Dry January, warmer than average winter
2014/2015	320.9	83.4	8.44	18.39	9	283	183	Dry, hot June
2015/2016	365.1	125.6	8.65	19.01	7	302	186	Late sowing, cold January, dry April
2016/2017	236.4	50.5	7.52	20.69	11	288	184	Cold January, dry grain-filling period
2017/2018	463.6	117.3	8.67	20.10	6	285	176	Cold January, wet March, dry April
2018/2019	349.4	117.0	8.92	20.27	13	277	183	Dry March and April, wet May
2019/2020	355.0	111.3	8.80	18.17	6	288	183	Dry March and April, cool year

Note: ^1^ Maximum daily temperature ≥30 °C; Σ = period from sowing to harvest; GFP = grain-filling period.

## Data Availability

Detailed meteorological data used in this study can be accessed through the Hungarian Meteorological Service. Home page: www.met.hu; e-mail: klimaker@met.hu.

## Supplementary Materials

The following supporting information can be downloaded at: https://www.mdpi.com/article/10.3390/plants11010113/s1, Table S1. Pearson’s correlation coefficients between meteorological factors and the gluten index and Minolta b* values of the five durum wheat genotypes over 16 years.
