# Supplementary material for: Effect of Multi-Year Environmental and Meteorological Factors on the Quality Traits of Winter Durum Wheat
Source: Plants (Basel). 2021 Dec 30;11(1):113. doi: 10.3390/plants11010113 (PMC8747632; doi:10.3390/plants11010113)
Supplement: Supplementary file 1 [file plants-11-00113-s001.zip › plants-1493029-supplementary.pdf]

Table S1. Pearson's correlation coefficients between meteorological factors and the gluten index and Minolta b* values of the five durum wheat genotypes over 16 years. *, ** and *** denote significant differences at the $p < 0.05$, 0.01 and 0.001 levels of probability.

Meteorological factors		GKB'		GKS'		GKM'		MVP'		MVH'		Cultivar Mean	
		GI	MB	GI	MB	GI	MB	GI	MB	GI	MB	GI	MB
AugR	Pearson r	0.091	0.056	-0.080	0.003	-0.127	0.174	0.179	0.191	0.078	-0.084	0.029	0.068
	sign.	0.739	0.838	0.767	0.992	0.639	0.520	0.506	0.480	0.774	0.757	0.916	0.801
SepR	Pearson r	-0.016	-0.342	-0.206	-0.438	0.049	-0.282	0.122	-0.296	0.118	-0.376	0.018	-0.367
	sign.	0.952	0.195	0.444	0.089	0.856	0.290	0.652	0.266	0.663	0.152	0.947	0.162
OctR	Pearson r	-0.371	-0.447	-0.126	-0.396	-0.342	-0.459	-0.017	-0.318	-0.216	-0.179	-0.270	-0.378
	sign.	0.157	0.082	0.641	0.129	0.195	0.073	0.952	0.231	0.421	0.506	0.311	0.149
NovR	Pearson r	0.438	0.214	0.332	0.092	-0.024	0.203	0.517*	0.234	0.395	0.324	0.370	0.226
	sign.	0.090	0.426	0.209	0.736	0.929	0.450	0.040	0.382	0.130	0.221	0.158	0.399
DecR	Pearson r	0.318	0.239	0.084	0.216	0.382	0.229	0.016	0.048	0.219	0.283	0.257	0.214
	sign.	0.230	0.373	0.757	0.421	0.144	0.394	0.952	0.859	0.416	0.289	0.336	0.425
JanR	Pearson r	-0.144	-0.162	0.040	-0.208	0.206	-0.187	-0.017	-0.282	-0.127	-0.169	-0.023	-0.212
	sign.	0.596	0.550	0.884	0.438	0.444	0.488	0.950	0.291	0.640	0.531	0.934	0.430
FebR	Pearson r	-0.178	-0.227	0.137	-0.222	-0.053	-0.224	-0.049	-0.274	-0.104	0.063	-0.071	-0.185
	sign.	0.510	0.398	0.614	0.408	0.846	0.404	0.857	0.305	0.702	0.816	0.793	0.494
MarR	Pearson r	-0.012	0.077	0.189	0.191	0.072	0.037	0.102	0.138	-0.040	0.122	0.055	0.120
	sign.	0.966	0.776	0.483	0.478	0.791	0.891	0.707	0.611	0.884	0.651	0.841	0.657
Apr1R	Pearson r	-0.462	-0.213	-0.467	0.112	-0.390	-0.073	-0.733	-0.046	-0.600	0.039	-0.589	-0.040
	sign.	0.071	0.429	0.068	0.680	0.135	0.787	0.001	0.866	0.014	0.887	0.016	0.884
Apr2R	Pearson r	0.205	0.095	0.326	0.119	-0.192	0.134	0.177	0.148	0.209	0.349	0.162	0.180
	sign.	0.447	0.726	0.218	0.661	0.477	0.620	0.512	0.583	0.437	0.186	0.549	0.506
Apr3R	Pearson r	0.167	0.287	0.215	0.272	-0.208	0.332	0.097	0.229	0.239	0.561*	0.123	0.356
	sign.	0.536	0.281	0.425	0.307	0.441	0.208	0.720	0.393	0.373	0.024	0.651	0.176
AprR	Pearson r	0.056	0.110	0.147	0.214	-0.313	0.193	-0.068	0.168	0.047	0.447	-0.025	0.240
	sign.	0.837	0.684	0.587	0.427	0.238	0.474	0.803	0.534	0.862	0.083	0.927	0.371
May1R	Pearson r	-0.433	-0.243	-0.491	-0.218	-0.363	-0.118	-0.451	-0.154	-0.394	-0.183	-0.479	-0.195
	sign.	0.094	0.364	0.054	0.418	0.167	0.663	0.079	0.568	0.131	0.498	0.060	0.469
May2R	Pearson r	-0.037	-0.155	-0.034	-0.063	-0.262	0.025	-0.175	0.018	-0.146	0.173	-0.145	-0.002
	sign.	0.890	0.568	0.900	0.818	0.328	0.927	0.517	0.948	0.590	0.522	0.592	0.996
May3R	Pearson r	-0.361	-0.062	-0.398	-0.032	-0.063	0.118	-0.517	-0.118	-0.433	-0.095	-0.392	-0.044
	sign.	0.170	0.819	0.127	0.905	0.817	0.663	0.040	0.664	0.094	0.727	0.133	0.872
MayR	Pearson r	-0.336	-0.208	-0.371	-0.129	-0.316	0.027	-0.497	-0.096	-0.422	0.000	-0.433	-0.089
	sign.	0.203	0.440	0.157	0.634	0.233	0.921	0.050	0.724	0.104	1.000	0.094	0.744
Jun1R	Pearson r	0.284	0.465	0.264	0.583*	-0.053	0.546*	0.129	0.617*	0.188	0.623*	0.191	0.597*
	sign.	0.286	0.069	0.323	0.018	0.845	0.029	0.634	0.011	0.486	0.010	0.479	0.015
Jun2R	Pearson r	-0.090	-0.111	-0.142	-0.131	-0.129	-0.194	0.020	-0.063	-0.151	-0.064	-0.122	-0.117
	sign.	0.739	0.682	0.599	0.628	0.635	0.472	0.941	0.816	0.577	0.813	0.652	0.666

Jun3R	Pearson r	0.205	-0.300	0.021	-0.404	0.566*	-0.236	-0.084	-0.418	0.269	-0.288	0.255	-0.349
	sign.	0.447	0.259	0.939	0.120	0.022	0.379	0.756	0.107	0.314	0.280	0.340	0.186
JunR	Pearson r	0.247	0.001	0.065	-0.012	0.246	0.027	0.042	0.058	0.178	0.150	0.195	0.048
	sign.	0.355	0.998	0.812	0.965	0.359	0.921	0.877	0.832	0.509	0.580	0.470	0.861
Jul1R	Pearson r	-0.009	0.158	0.291	0.104	-0.303	0.202	0.322	0.040	0.144	0.158	0.073	0.139
	sign.	0.975	0.558	0.274	0.701	0.254	0.453	0.225	0.884	0.594	0.560	0.787	0.609
Jul2R	Pearson r	-0.128	-0.337	-0.197	-0.331	-0.164	-0.417	-0.078	-0.273	-0.062	-0.116	-0.142	-0.308
	sign.	0.636	0.203	0.464	0.211	0.543	0.108	0.774	0.307	0.819	0.668	0.601	0.247
Jul12R	Pearson r	-0.123	-0.254	-0.050	-0.271	-0.301	-0.306	0.078	-0.246	0.011	-0.040	-0.099	-0.233
	sign.	0.651	0.343	0.854	0.309	0.257	0.248	0.773	0.358	0.967	0.883	0.716	0.385
SumDEV55R	Pearson r	-0.106	-0.072	-0.207	-0.066	0.024	0.071	-0.291	-0.057	-0.204	0.017	-0.166	-0.024
	sign.	0.695	0.790	0.441	0.809	0.929	0.793	0.273	0.835	0.449	0.949	0.539	0.928
SumAugR	Pearson r	-0.015	-0.193	-0.039	-0.199	-0.100	-0.055	0.026	-0.104	-0.012	0.056	-0.035	-0.106
	sign.	0.956	0.473	0.885	0.459	0.712	0.840	0.924	0.701	0.964	0.838	0.898	0.697
SumR	Pearson r	0.016	-0.017	0.071	0.038	-0.058	0.106	-0.079	0.020	-0.056	0.300	-0.023	0.094
	sign.	0.954	0.950	0.793	0.888	0.831	0.697	0.771	0.942	0.838	0.259	0.934	0.729
OktT	Pearson r	-0.149	0.596*	-0.046	0.416	-0.100	0.481	-0.002	0.445	-0.184	0.495	-0.126	0.515*
	sign.	0.582	0.015	0.864	0.109	0.713	0.059	0.995	0.084	0.494	0.051	0.642	0.041
NovT	Pearson r	-0.003	0.035	0.115	-0.254	0.053	-0.095	0.391	-0.008	0.035	-0.016	0.102	-0.068
	sign.	0.990	0.897	0.671	0.343	0.845	0.727	0.134	0.976	0.899	0.952	0.707	0.801
DecT	Pearson r	-0.105	0.156	-0.160	-0.001	0.123	0.008	-0.011	-0.070	0.000	0.085	-0.033	0.040
	sign.	0.700	0.563	0.554	0.998	0.650	0.977	0.968	0.797	1.000	0.754	0.902	0.883
JanT	Pearson r	-0.101	0.369	-0.152	0.483	-0.225	0.392	-0.059	0.497	-0.182	0.322	-0.170	0.435
	sign.	0.710	0.159	0.575	0.058	0.403	0.134	0.828	0.050	0.501	0.224	0.529	0.093
FebT	Pearson r	-0.212	0.105	-0.045	-0.084	-0.149	-0.043	0.174	0.114	-0.222	-0.133	-0.139	-0.007
	sign.	0.430	0.699	0.868	0.756	0.582	0.873	0.520	0.674	0.409	0.623	0.608	0.979
MarT	Pearson r	-0.114	-0.177	-0.254	-0.247	-0.161	-0.181	-0.074	-0.133	-0.084	-0.430	-0.154	-0.248
	sign.	0.673	0.512	0.342	0.357	0.551	0.502	0.786	0.622	0.756	0.097	0.570	0.354
Apr1T	Pearson r	0.356	-0.197	0.032	-0.356	0.348	-0.253	0.197	-0.210	0.463	-0.165	0.346	-0.248
	sign.	0.175	0.465	0.906	0.175	0.186	0.343	0.465	0.436	0.071	0.542	0.189	0.355
Apr2T	Pearson r	0.046	0.232	0.085	0.170	0.274	0.092	0.131	0.148	0.091	0.316	0.138	0.205
	sign.	0.864	0.388	0.753	0.530	0.304	0.735	0.630	0.586	0.738	0.234	0.609	0.446
Apr3T	Pearson r	-0.060	0.188	-0.041	0.326	0.163	0.310	-0.258	0.162	-0.158	0.167	-0.070	0.241
	sign.	0.826	0.485	0.879	0.218	0.547	0.243	0.334	0.549	0.560	0.537	0.796	0.370
AprT	Pearson r	0.206	0.100	0.034	0.054	0.442	0.072	0.026	0.036	0.234	0.146	0.236	0.087
	sign.	0.445	0.712	0.901	0.843	0.086	0.790	0.922	0.895	0.383	0.590	0.378	0.748
May1T	Pearson r	-0.342	-0.216	-0.143	0.020	-0.112	-0.175	-0.231	-0.147	-0.305	-0.106	-0.269	-0.132
	sign.	0.195	0.421	0.597	0.943	0.679	0.516	0.390	0.588	0.251	0.696	0.313	0.626
May2T	Pearson r	0.347	0.249	0.444	0.132	0.477	0.117	0.432	0.115	0.439	0.010	0.482	0.132
	sign.	0.189	0.353	0.085	0.626	0.062	0.665	0.095	0.673	0.089	0.970	0.059	0.625
May3T	Pearson r	0.232	-0.038	0.061	0.044	-0.053	-0.024	0.106	0.180	0.282	0.109	0.160	0.058
	sign.	0.387	0.888	0.822	0.872	0.845	0.929	0.696	0.505	0.290	0.688	0.555	0.832

MayT	Pearson r	0.100	-0.038	0.154	0.099	0.116	-0.064	0.125	0.078	0.194	0.010	0.158	0.019
	sign.	0.713	0.890	0.568	0.716	0.669	0.815	0.644	0.774	0.472	0.970	0.558	0.945
Jun1T	Pearson r	-0.347	-0.431	-0.502	-0.400	-0.315	-0.390	-0.050	-0.190	-0.211	-0.607	-0.335	-0.428
	sign.	0.188	0.096	0.048	0.125	0.234	0.135	0.853	0.481	0.433	0.013	0.205	0.098
Jun2T	Pearson r	-0.535	0.190	-0.409	0.177	-0.177	0.282	-0.623	0.199	-0.586	0.065	-0.528	0.190
	sign.	0.033	0.481	0.116	0.513	0.513	0.290	0.010	0.460	0.017	0.810	0.035	0.481
Jun3T	Pearson r	-0.107	0.229	-0.192	0.255	-0.159	0.204	-0.353	0.251	-0.197	0.135	-0.213	0.226
	sign.	0.693	0.394	0.475	0.340	0.557	0.449	0.180	0.348	0.465	0.618	0.429	0.399
JunT	Pearson r	-0.556	-0.054	-0.660	-0.021	-0.403	-0.001	-0.557	0.129	-0.546	-0.296	-0.615	-0.054
	sign.	0.025	0.843	0.005	0.938	0.121	0.996	0.025	0.634	0.029	0.266	0.011	0.843
Jul1T	Pearson r	-0.517	-0.364	-0.616	-0.164	-0.223	-0.298	-0.592	-0.369	-0.498	-.546*	-0.545	-0.371
	sign.	0.040	0.166	0.011	0.543	0.407	0.262	0.016	0.159	0.050	0.029	0.029	0.158
Jul2T	Pearson r	0.403	0.070	0.434	0.011	0.331	0.247	0.264	0.070	0.383	0.069	0.421	0.095
	sign.	0.122	0.796	0.093	0.969	0.210	0.356	0.323	0.797	0.143	0.799	0.104	0.726
Jul12T	Pearson r	-0.082	-0.236	-0.136	-0.125	0.095	-0.036	-0.258	-0.240	-0.084	-0.381	-0.090	-0.220
	sign.	0.762	0.379	0.614	0.646	0.728	0.894	0.334	0.371	0.758	0.145	0.740	0.413
DEV55T	Pearson r	-0.052	-0.011	-0.208	-0.029	-0.084	0.071	-0.190	0.208	-0.058	-0.126	-0.120	0.022
	sign.	0.847	0.969	0.439	0.914	0.756	0.794	0.481	0.439	0.830	0.641	0.658	0.936
SumT	Pearson r	-0.157	0.150	-0.185	-0.041	-0.034	0.081	0.063	0.150	-0.142	-0.062	-0.118	0.059
	sign.	0.562	0.579	0.492	0.881	0.901	0.765	0.815	0.580	0.600	0.819	0.663	0.828
May1H	Pearson r	-0.050	0.006	-0.191	0.228	-0.244	0.009	-0.280	0.063	-0.073	0.222	-0.170	0.112
	sign.	0.855	0.983	0.480	0.396	0.363	0.974	0.293	0.818	0.788	0.408	0.528	0.678
May2H	Pearson r	0.533*	0.248	0.376	-0.001	0.859**	0.006	0.352	-0.010	0.511*	0.102	0.617*	0.077
	sign.	0.034	0.354	0.151	0.998	0.000	0.982	0.182	0.969	0.043	0.708	0.011	0.778
May3H	Pearson r	0.273	0.671**	0.266	0.626**	0.026	0.568*	0.174	0.777**	0.123	0.609*	0.195	0.688**
	sign.	0.306	0.004	0.319	0.010	0.924	0.022	0.518	0.000	0.649	0.012	0.470	0.003
MayH	Pearson r	0.333	0.617*	0.252	0.586*	0.146	0.477	0.145	0.663**	0.197	0.593*	0.253	0.622*
	sign.	0.207	0.011	0.346	0.017	0.589	0.062	0.591	0.005	0.464	0.015	0.344	0.010
Jun1H	Pearson r	-0.006	0.146	0.069	0.058	-0.240	0.231	0.222	0.266	0.029	-0.074	-0.002	0.129
	sign.	0.981	0.590	0.799	0.831	0.371	0.389	0.409	0.319	0.916	0.786	0.993	0.633
Jun2H	Pearson r	-0.304	0.412	-0.256	0.396	0.061	0.483	-0.544	0.345	-0.423	0.150	-0.321	0.374
	sign.	0.253	0.112	0.338	0.129	0.823	0.058	0.029	0.190	0.102	0.579	0.225	0.154
Jun3H	Pearson r	0.045	0.355	0.039	0.403	-0.081	0.330	-0.203	0.379	-0.068	0.260	-0.048	0.364
	sign.	0.867	0.177	0.886	0.122	0.766	0.212	0.450	0.148	0.801	0.331	0.859	0.166
JunH	Pearson r	-0.170	0.520*	-0.115	0.503*	-0.099	0.583*	-0.378	0.536*	-0.298	0.215	-0.233	0.494
	sign.	0.528	0.039	0.672	0.047	0.716	0.018	0.149	0.032	0.263	0.424	0.385	0.052
Jul1H	Pearson r	-0.273	-0.151	-0.285	-0.048	0.012	-0.136	-0.309	-0.220	-0.308	-0.464	-0.262	-0.218
	sign.	0.307	0.577	0.284	0.860	0.964	0.615	0.245	0.412	0.245	0.070	0.327	0.417
Jul2H	Pearson r	0.538*	0.237	0.505*	0.182	0.368	0.407	0.248	0.185	0.412	0.164	0.487	0.244
	sign.	0.032	0.377	0.046	0.501	0.161	0.118	0.354	0.492	0.113	0.543	0.056	0.362
Jul12H	Pearson r	0.174	0.057	0.145	0.088	0.250	0.178	-0.039	-0.023	0.068	-0.196	0.148	0.017

	sign.	0.518	0.835	0.593	0.746	0.351	0.510	0.885	0.934	0.802	0.466	0.585	0.949
SumH	Pearson r	0.166	0.662**	0.111	0.611*	0.080	0.728**	-0.195	0.703**	-0.053	0.483	0.042	0.670**
	Szignifikancia	0.539	0.005	0.682	0.012	0.768	0.001	0.468	0.002	0.845	0.058	0.877	0.005

Abbreviations for meteorological factors: 3-letter month code + 1, 2, 3 (1st, 2nd, 3rd 10-day period) or nothing in the case of the whole month + R = rainfall, T = mean temperature, H = No. of heat days.

SumDEV55R: total precipitation from DEV55 (heading).

Sum AugR: total precipitation from August 1.

SumR: total precipitation from sowing to harvest.

DEV55T: average temperature from heading.

SumT: average temparature in the whole growing season

SumH: total number of heat day in the whole growing season.
